# Effects of sonication on particle dispersions from a size, biodissolution, cytotoxicity and transferred dose perspective – a case study on nickel and nickel oxide particles

**DOI:** 10.1371/journal.pone.0323368

**Published:** 2025-05-09

**Authors:** Eva Blomberg, Xuying Wang, Gunilla Herting, Alexander Khort, Abishek Arora, Samuel Buxton, Tara Lyons-Darden, Hanna L. Karlsson, Inger Odnevall

**Affiliations:** 1 KTH Royal Institute of Technology, School of Engineering Sciences in Chemistry, Biotechnology and Health, Department of Chemistry, Division of Surface and Corrosion Science, Stockholm, Sweden; 2 Institute of Environmental Medicine, Karolinska Institutet, Stockholm, Sweden; 3 NiPERA. Inc.,2525 Meridian Parkway, Suite240, Durham, N.C., United States of America; 4 AIMES–Center for the Advancement of Integrated Medical and Engineering Sciences at Karolinska Institutet and KTH Royal Institute of Technology, Stockholm, Sweden; 5 Karolinska Institutet, Department of Neuroscience, Stockholm, Sweden; Mass Eye Infirmary, Harvard Medical School / Northeastern University, UNITED STATES OF AMERICA

## Abstract

The toxicity of micro- and nanoparticles in cell culture studies is influenced by factors like particle size, agglomeration, dissolution of the particles, and methodological factors like sonication protocols. The main aim of this study was to investigate the influence of sonication on the particle size, dissolution, cytotoxicity, and dosing accuracy of nickel (Ni) metal and Ni oxide (NiO) particle dispersions. Such investigations are important to enable studies on the cellular uptake of different Ni substances in lung cells. The effect of sonication was evaluated in ultrapure water, two types of cell media, and A549 human lung cells using the tip and water bath methods. Extended sonication significantly decreased particle size and increased particle dissolution, emphasizing the need for optimized sonication conditions tailored to the specific particle type and study design. Observed findings demonstrate that the sonication step potentially can have a large impact on the results due to changes in particle characteristics, size, and dissolution, properties which are highly dependent on the particle type, solution composition, and sonication parameters. Although only small differences were observed in the limited assessment of cytotoxicity (A549 cells) in this study, further investigation is required to determine the impact of sonication on toxicity. This study also emphasizes the need to evaluate transferred dose samples due to the evident effects of agglomeration, sedimentation, and losses during sample transfer of particle dispersions. The study clearly illustrates that the choice of sonication protocol is particularly critical for toxicity studies, which are the basis of government regulatory decisions and human exposure limits.

## Introduction

A comprehensive understanding of interactions between micron- and nanosized metallic particles and human and environmental systems across various societal applications and occupational settings is essential for assessing potential risks and ensuring their safe handling and use. Such assessments include *in vitro* testing using particle dispersions, which often are prepared from non-functionalized particles in their dry state. Preparation of metallic particle dispersions, and understanding of their characteristics in terms of colloidal and chemical stability and transferred (administrated versus nominal) dose need to be assessed and considered prior to and during *in vitro* test conditions since these are critical factors that shape the interaction (for example, uptake, toxicity) between the particles and targeted biological systems [1]. The importance of these and other aspects (e.g., aging effects, presence of capping agents, chemical speciation) for metallic particles of different sizes have been elucidated in the literature [2,3].

Due to their high surface energy, non-functionalized metallic micron- and nanosized particles tend to readily agglomerate in suspension, a process which is highly metal and solution (e.g., chemical composition, pH, ionic strength) dependent. Sonication is widely employed when preparing solutions of metallic particle dispersions to break up particle agglomerates, promote stable dispersions and minimize further agglomeration [4]. Sonication using a tip or water bath or mixing by vortexing are common ways to prepare particle dispersions for *in vitro* and *in vivo* toxicity testing [4,5]. Since particle stability and extent of agglomeration/aggregation are strongly particle (metal, dose, etc.) and solution-dependent, these aspects need to be considered and evaluated in toxicity studies.

The applied energy and duration of sonication are key factors that influence not only the particle size distribution and particle stability in solution, but also the overall performance of the particles. Recent literature highlights the importance of optimizing sonication parameters, such as time, frequency, and power intensity, to ensure the desired dispersion without causing excessive particle fragmentation or surface modification [5–7]. Prolonged sonication, while effective for deagglomeration, may lead to changes in particle properties, including increased biodissolution and/or alterations in surface chemistry. This can influence the particle dose and bioavailability by modifying the particle surface area and reactivity, which in turn can influence their toxic potency and/or environmental behavior [8–10].

Protocols for preparing particle dispersions, e.g., stock solutions, of non-functionalized dry metallic particles using sonication, such as the Nanogenotox protocol and OECD technical guidance documents, emphasize the need for careful control of sonication parameters, along with the choice of solvent, temperature regulation, and post-sonication validation techniques, such as dynamic light scattering (DLS) and zeta potential measurements [11–15]. These documents typically propose ultrasonic treatments by means of using tip (probe) sonication with a defined transferred acoustic energy and sonication time with the aim to gain stable dispersions for up to 1 h without considering agglomeration and sedimentation effects [11–15]. The sonication time is especially critical, as it determines the efficiency of deagglomeration and ensures the stability and homogeneity of the dispersion. Optimal sonication time prevents re-aggregation and overheating, while avoiding excessive particle breakdown, biodissolution and changes in surface characteristics already in the stock solution that could alter the desired particle properties to be investigated [13]. The Nanogenotox protocol stipulates for example, preparation of stock solutions using ultrapure water, including 0.05 wt.% bovine serum albumin (BSA) for particle stabilization purposes, and sonication time periods up to 12–16 minutes, depending on the probe amplitude settings [15]. However, such long sonication treatments may, depending on the particle properties, result in a large fraction of particle dissolution (release of metals) already in the stock solution, as well as influence the surface characteristics. Increased dissolution of particle dispersions by the presence of BSA has previously been reported for Cu, Al, Mn, Ag, ZnO and CuO nanoparticles (NPs) [9,16,17]. In addition, literature findings have shown that a sonication treatment of aqueous solutions containing BSA can damage the BSA conformation, as well as promote particle dissolution via ligand-induced metal release processes [4,18,19].

As described above, available protocols for preparing particle dispersions, including metallic particles, using sonication focus on optimizing sonication conditions, selecting appropriate solvents and stabilizers, controlling temperature, and validating particle dispersion quality to ensure consistent and reproducible solutions [13,14]. Sonication is crucial for controlling particle dispersion, stability, and performance, particularly for particle research and chemistry research. However, in toxicological studies, especially with NPs that tend to readily agglomerate, studies incorporating ‘sufficient’ particle dispersion likely do not generate data that appropriately represent human exposure [20–22]. It is hence quite important to clarify the ultimate purpose of the research and potential impacts of sonication before optimizing the protocol for each type of particle to ensure the integrity of the study.

This study investigates the impact of sonication time on the size, dissolution, and transferred dose of Ni and NiO particle dispersions of varying sizes in both ultrapure water and cell culture media, following the general procedures of particle dispersion preparations for toxicity testing [4]. This was initiated as a preliminary inquiry prior to a more extensive investigation of the cellular uptake of different Ni and NiO particles in cell culture conditions. Micron- and nanosized Ni and NiO particles have many commercial and industrial uses in electronics, batteries, paints, transportation products, as well as many other applications. Potential health and environmental exposure to Ni and NiO particles from such wide-ranging applications are often the basis for their continued evaluation in many biological research studies, thus relevant for this case study evaluating the effects of sonication in these types of studies [23–25].

The first objective of this study was to evaluate how extended sonication time influences particle size and dissolution across different micron- and nano-sized particles. Secondly, the study aimed to determine whether longer sonication time of stock solutions containing micron- and nano-sized Ni particles alters the cytotoxic response in a cellular assay. Thirdly, the effect of sonication time on the transferred dose of these particles was analyzed. Underlying theoretical reasons for the strong tendency of agglomeration of metallic particles in solution and its challenges for particle research are shortly discussed.

## Materials and methods

### Investigated particles

The test particles were Ni metal micron-sized powders (Ni, CAS# 7440-02-0, Sumitomo, Tokyo, Japan), NiO micron-sized powders (CAS# 1313-99-1, Umicore, Brussels, Belgium), as well as nano-sized particles of two different sizes (80 and 20 nm, based on supplier information) of Ni metal (Ni80 NPs and Ni20 NPs, Miyou, Suzhou, China) and NiO (NiO80 NPs and NiO20 NPs, Nanoshel, LLC, Wilmington, DE, USA). Soluble Ni sulfate hexahydrate (NiSO_4_ × 6H_2_O, CAS# 10101-97-0, Sigma Aldrich, USA) was included for comparison. The tip sonication study in ultrapure water was conducted on NPs of Ni metal (CAS# 7440-02-0, purity >99%, based on supplier information) and NiO (CAS# 1313-99-1, purity 99.8%, based on supplier information) from Sigma Aldrich (Sweden).

### Equipment cleaning procedures

All equipment in contact with solutions intended for trace Ni analysis was acid-cleaned by 10 vol.% HNO_3_ (ultrapure quality, VWR, Sweden) for at least 24 h at room temperature, followed by rinsing with ultrapure water at least four times and drying at ambient conditions and room temperature prior to use. Tweezers and spoons were cleaned with 95% ethanol and ultrapure water and dried with paper towels between handling different substances. Ultrapure water was used throughout this study (resistivity – 18.2 MΩcm, Millipore, Solna, Sweden).

### Exposure media

Results are presented for three different exposure media; i) ultrapure water, ii) DMEM + , Dulbeccos´s Minimal Essential Medium with serum, the same medium as used to culture the A549 cells, see below (10% Fetal Bouvine Serum (FBS), 1 mM sodium pyruvate and 1% penicillin-streptomycin from Gibco), and iii) Bronchial epithelial cell growth medium (BEGM, Lonza), supplemented with BEGM BulletKit, the same medium as used to culture the human bronchial epithelial cell line (BEAS-2B, European Collection of Cell Cultures) [26].

### Particle characterization at dry conditions

Particle morphology and the extent of aggregation were investigated at dry conditions for assemblies of particles mounted on carbon tape by means of Field Emission Scanning Electron Microscopy (FEI XL30, Thermo Fisher, Germany) operating at 15 kV using secondary electrons.

### Chemical analysis of Ni in solution

Atomic absorption spectroscopy (AAS), using a PinAAcle 900T instrument (Perkin Elmer), was used in either graphite furnace (GF) mode (µg/L concentrations) or flame mode (F) (mg/L concentrations) to determine released and total concentrations of Ni in solution. Blank samples were prepared and measured for each exposure condition and subtracted from the measurement of the different powder samples to remove background contaminations, if any. All samples were acidified to a pH below 2 (using ultrapure 65% HNO_3_) for total Ni concentration analysis. Calibration was performed using standards of known concentrations (0, 10, 30, 100 µg/L (GF), and 0, 4, 10, 30 mg/L (F)). Quality control samples were run every 6th sample. The limits of detection were 0.6 µg/L (GF) and 20 µg/L (F), and the limits of quantification were 1.5 µg/L (GF) and 50 µg/L (F).

### Sonication of stock solutions and dose samples

Stock solutions of Ni MPs and Ni80 NPs (1 mg Ni/mL) were prepared in cell media (BEGM or DMEM+) followed by sonication in a water bath (VWR, Sweden) for 1, 5 or 15 min at room temperature. Dose samples were prepared by pipetting 1 mL of the stock particle suspension to a new vessel diluted with 9 mL fresh medium to generate a Ni loading of 0.1 mg/mL for each particle solution.

Stock solutions of Ni or NiO NPs (Sigma, Sweden) (100 mg/L) were prepared in ultrapure water by dispersing 1 mg in 10 mL of ultrapure water in acid-cleaned glass vials, being tip sonicated (Q-Sonica, Q500 (USA), constant mode, amplitude 20%, tip diameter 6 mm) every 2^nd^ min up to 16 min. The selected times are based on the Nanogenotox protocol stipulating sonication for 12 or 16 min [15].

### Biodissolution - effect of sonication time

**Fig 1:**
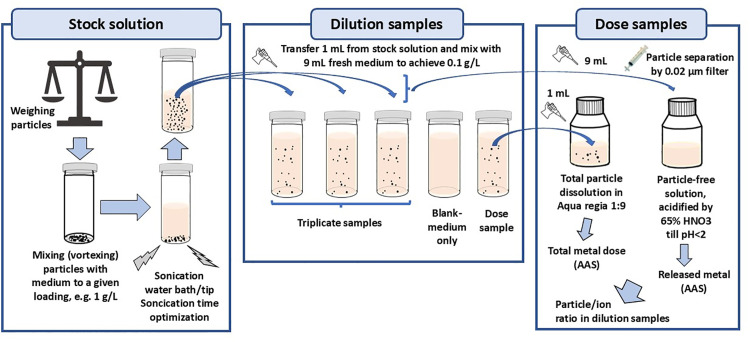
Schematic illustration of the experimental approach employed in this study for preparing particle stock solutions, dilution samples and dose samples for further biodissolution and cytotoxicity investigations.

The general experimental procedures for preparing particle stock solutions, dilution samples and dose samples for further biodissolution and cytotoxicity studies and effects of sonication time are illustrated in Fig 1. This experimental setup is intentionally different from the standard biodissolution testing (direct preparation of particle concentrations of interest without successive dilutions), since we aimed to mimic the same experimental approach employed when performing cellular toxicity studies in terms of stock solution preparation, sonication, dilutions, etc.

Stock solutions (1 mg Ni/mL) were prepared in BEGM, followed by sonication in a water bath (WVR, Sweden) for 1, 5 or 15 min (for Ni MPs and Ni80 NPs) at room temperature. Dose and dilution samples were prepared by pipetting 1 mL of the stock particle suspension to a new vessel and diluted with 9 mL of fresh medium to generate a Ni loading of 0.1 mg/mL for each particle solution. After the different sonication times, 9 mL of the solution samples were collected from which the powder particles were removed using an alumina-based syringe filters with a pore size of 20 nm (Anotop 25, Whatman, USA), determining the extent of released Ni immediately after the different sonication times. The remaining 1 mL was collected, including settled agglomerates and non-dissolved particles, for complete dissolution in diluted Aqua regia (2.5 vol.% HCl (pro analysi, VWR, Sweden) and 6.5 vol.% HNO_3_ in ultrapure water, pH < 0.1) to determine the transferred dose for each time-period investigated. All solution samples were stored in acid-cleaned new vessels and analyzed for total Ni concentrations by means of AAS as described above.

NP stock solutions (100 mg/L) for the sonication study in ultrapure water were prepared by dispersing 1 mg of Ni or NiO NPs (from Sigma, Sweden) in 10 mL of ultrapure water in acid-cleaned glass vials by means of tip sonication (see info above) every 2^nd^ min up to 16 min. 1 mL of the stock solution was immediately transferred into 15 mL Falcon tubes, diluted with 9 mL ultrapure water, and vortexed for 1 min, obtaining solutions with the nominal concentration of 10 mg/L. One tube was used for dose analysis, whereas other used to study changes in NP concentration and particle size for the different sonication times. The extent of Ni dissolution from the particles were determined immediately after the sonication step after removing undissolved NPs by using 20 nm Anotop syringe filters. The remaining solution was acidified and the total amount of released Ni determined by means of AAS as described above.

### Particle size and concentration - effect of sonication time

Changes in particle size distribution and extent of particle settling directly after different sonication times were investigated for the Ni MPs and the Ni80 NPs in cell medium by means of dynamic light scattering using photon cross-correlation spectroscopy (PCCS) (NanoPhox, Sympatec, Germany). All solution samples (nominal concentration 1 mg/L) were prepared in UV-cuvettes (routine pack, Sympatec GmbH, Claustal, Germany). Standard latex samples (100 nm ± 10 nm) were tested prior to analysis to ensure the accuracy of the measurements.

Nanoparticle Tracking analysis (NTA), using a NanoSight N300 (Malvern, UK) instrument equipped with a Blue 405 nm laser, and sCMOS camera, was employed to determine the hydrodynamic particle size and concentration distribution of suspensions of Ni and NiO NPs (from Sigma, nominal concentration 10 mg/L) in ultrapure water directly after different times of tip sonication (every 2^nd^ min up to 16 min). Five videos each 60 seconds were acquired for each sample, and three individual samples were prepared for each time point. The camera level was set to 7 or 8 and adjusted in every individual case for better particle visibility. The results were analysed using the NTA software. A detection threshold of 10 was applied for all videos. The hydrodynamic diameters were determined using the Stokes-Einstein equation [27]. The extent of particle dissolution as a function of different sonication times was determined by means of AAS as described above.

### Cytotoxicity – effect of sonication time

To determine the effect of sonication time (1 and 15 min) on particle toxicity, the Alamar blue assay (Invitrogen) was performed. A549 cells, originating from an adenocarcinoma in human alveolar basal epithelial cells of a 58-year-old Caucasian male (from the American Type Culture Collection) were seeded in 96 well plates (Corning) with a seeding density of 10,000 cells/well and placed in an incubator set at 5.0% CO_2_, 95% air at 37°C. The exposures were started the day after seeding. In brief, 1 mg/mL stock suspensions of Ni MPs and Ni80 NPs were prepared in DMEM+ medium followed by sonication for 1 min (ultrasonic bath) and immediate preparation of final exposure concentrations (5.0, 25.0, 50.0, 75.0, 87.5, 100.0 and 150.0 µ L/mL). The same stock was then sonicated for another 14 min (total sonication time: 15 min), followed by preparation of the exposure concentrations. As a positive control for the assay, cells were treated with 10% dimethyl sulfoxide (DMSO, Sigma, Sweden). First, experiments were performed for a broad range of concentrations (5.0, 25.0, 50.0, 75.0, 87.5, 100.0 and 150.0 µ L/mL), with cells exposed for 24 and 48 h, three wells per exposure in two independent experiments. Next, cells were exposed to a narrower concentrations range, based on the estimated steepest part of the dose-response curve (10.0, 25.0, 40.0 and 50.0 µ L/mL), five wells per exposure concentrations in two independent experiments.

Following the exposure periods, 10% of Alamar blue (Invitrogen) solution was prepared in DMEM+ medium. The exposure medium was aspirated followed by the addition of the 10% Alamar blue solution (100 µ L/well) for 2 h. The fluorescence was then recorded using a microplate reader at Ex 540 nm/Em 590 nm (Tecan Infinite F 200 Pro, Magellan v7.2). The readings were blank corrected, and the fluorescence values were normalized to the mean fluorescence of the negative control, representing 100% cell viability. The data analysis was performed in R (v4.3.2) using two-way ANOVA (GraphPad) followed by Šídák’s multiple comparisons test.

## Results and discussion

### Effects of tip sonication time on particle dispersions in ultrapure water from a particle size, particle concentration and dissolution perspective

**Fig 2:**
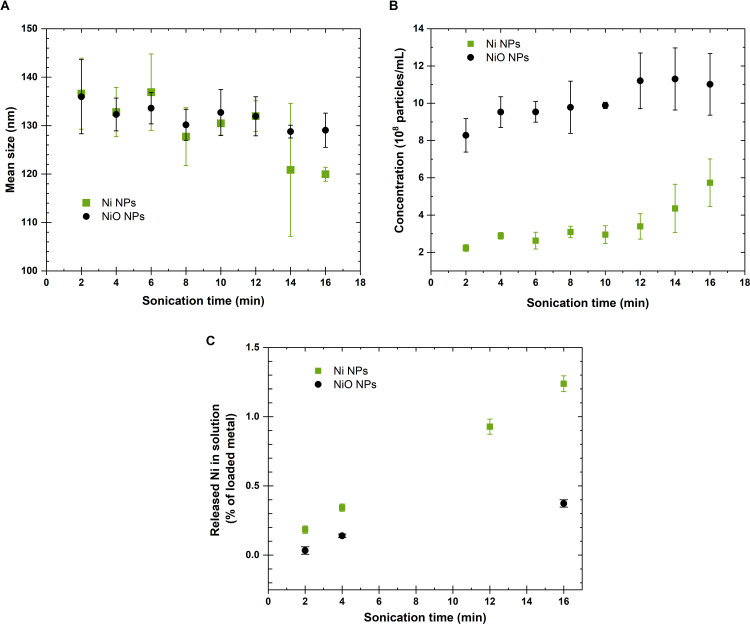
Effect of tip sonication time on particle size and dissolution of Ni and NiO NPs in ultrapure water. Changes in mean particle size (A) and concentration (B) of Ni NPs (squares) and NiO NPs (circles) (three independent samples of each powder) determined by means of NTA in ultrapure water stock solutions (100 mg/L powder) tip-sonicated for different time periods (2, 4, 6, 8, 10, 12, 14 or 16 min), and(C) corresponding released fractions of Ni (% of the transferred mass of Ni based on dose sample measurements) measured directly after each sonication time period.

The effect of different tip sonication times was investigated for Ni and NiO NPs (100 mg/L) in ultrapure water. Kinetic results of changes in particle size distribution and concentration, measured every 2^nd^ min up to 16 min, are presented in Figs 2A and 2B for the Ni and NiO NPs nominally sized as 100 nm (at dry conditions varying between 20 and 200 nm based on TEM measurements) [28]. The mean hydrodynamic particle size in solution decreased with sonication time (Fig 2A) showing a greater size reduction with increased sonication time for the Ni NPs compared to the NiO NPs. After 14–16 min of sonication, the reduction was approximately three times higher for the Ni NPs compared to the NiO NPs. Both powders showed an increased particle number concentration in solution with increased sonication time (Fig 2B). The results demonstrate that the tip sonication process, at least to some extent, can separate some of the agglomerated particles, and/or that larger sized particles/agglomerates have settled, leaving the smaller sized particles/agglomerates in solution. Similar findings have been observed for Cu, Mn and Al NPs being tip-sonicated for 3 and 15 min in 1 mM NaClO_4_ [9].

An extended time of tip sonication of the Ni and NiO NP stock solutions (100 mg particles/L) increased the extent of released Ni from both powders (measured directly after the sonication step). Approximately 3 times more Ni was released per mass from the Ni NPs compared to the NiO NPs

(Fig 2C). This observation is comparable to the kinetics of the mean particle size change, while the kinetics in particle concentration kinetics was ~ 12% higher for the NiO NPs. These findings suggest that the faster reduction of the particle size of the Ni NPs compared to the NiO NPs primarily. These results, with partial particle dissolution taking place already in the stock solution and reduced particle size with increased sonication time are in line with the scarce literature available on the topic for metallic particles. 2–5% dissolution of the particle mass has for example been shown to take place during tip-sonication (15 min) of stock solutions of Cu, CuO, ZnO, Al and Mn NPs [16–18].

Overall, tip sonication of the Ni and NiO NP stock solutions in ultrapure water clearly increased the extent of particle dissolution, decreased the particle size distribution and increased the particle concentration in solution. These effects increased with increased tip sonication time.

### Effects of water bath sonication time on particle dispersions in cell media from a particle size, density distribution and dissolution perspective

**Fig 3:**
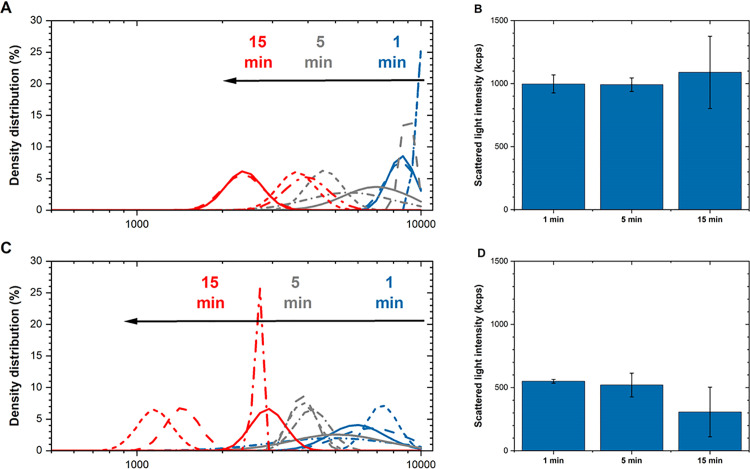
Effect of water bath sonication time on particle size distribution and scattered intensity for Ni MPs and Ni80 NPs in cell medium. Changes in hydrodynamic particle size distributions of micron- (**A**, Ni MPs) and nano-sized (**C**, Ni80 NPs) particles in cell medium (DMEM+) sonicated for 1, 5 or 15 min in a water bath. Data is presented as the density distribution by volume with corresponding scattered light intensity (**B**, Ni MPs and **D**, Ni80 NPs). Four independent samples (solid and dotted lines of the same color) were investigated for each sonication time point and particle type.

The effect of sonication time on the particle size and extent of dissolution from nano-sized (Ni80 NPs) and micron-sized (Ni MPs) Ni particles in DMEM+ cell medium stock solution were investigated using a sonication water bath treatment as this is a commonly used methodology to prepare particle suspensions for, e.g., toxicological dose-response investigations. The study included three sonication times (1, 5 or 15 min). Changes in particle size distributions with increased time of sonication are presented in Figs 3A and 3C for Ni MPs and Ni80 NPs, respectively) based on four separate PCCS measurements of each particle type and sonication time together with corresponding changes in mean scattered light intensities which is a measure of particle settling (Figs 3B and 3D). Changes in mean particle sizes (X_50_) in DMEM+ are presented in Table 1.

**Table 1:** Effect of water bath sonication time on the mean particle size of Ni MPs and Ni80 NPs in cell medium. Mean particle sizes, X50, determined from DLS measurements (quadruple measurements of each exposure condition) of stock solutions of Ni MPs and Ni80 NPs dispersed in cell medium (DMEM+) sonicated for 1, 5 or 15 min in a water bath.

Ni MPs			
**Sonication time**	**1 min**	**5 min**	**15 min**
**X**_**50**_ **(nm)**	9193 ± 838	6460 ± 1913	3085 ± 833
**Ni 80 NPs**			
**Sonication time**	**1 min**	**5 min**	**15 min**
**X**_**50**_ **(nm)**	6121 ± 1108	4202 ± 554	2046 ± 874

Despite substantial variations in particle size distributions for the Ni MP and the Ni80 NPs (Figs 3A and 3C), the scattered light intensity remained relatively consistent with increasing sonication time for the Ni MPs, while the Ni80 NPs exhibited twice as low scattered intensity, and a trend towards reduced levels, in particular after 15 min. However, even though this trend was not statistically significant due to the high variability between measurements (see Table 1), these results reflect the shift towards smaller particle size distributions of the Ni80 NPs after 5 min. The effect was most evident after 15 min of sonication, most probably as a combined result of settling of larger-sized particles and agglomerates as well as deagglomeration into smaller particle sizes, with lower scattered light intensity for the smaller-sized particles/agglomerates as a result.

**Fig 4:**
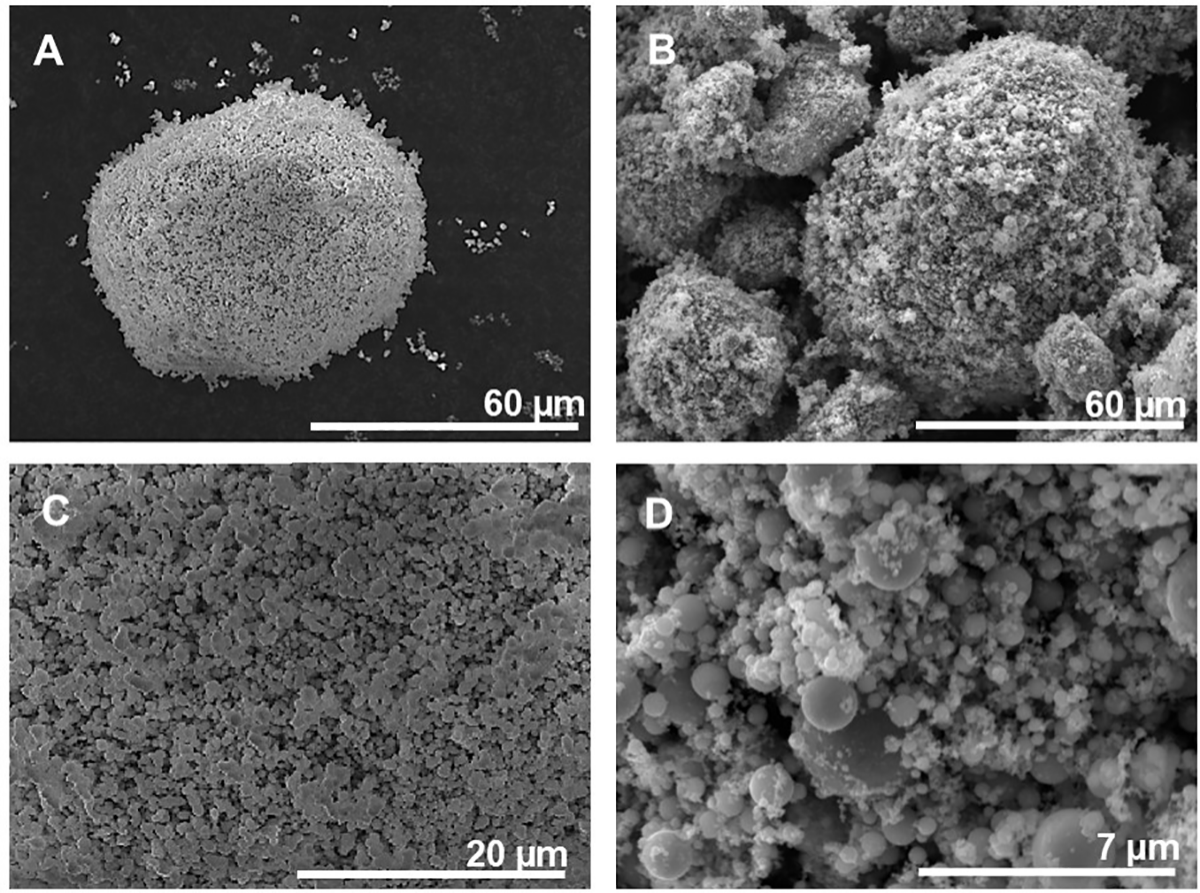
Particle morphology of Ni MPs and Ni80 NPs at dry conditions. Scanning electron microscopy, SEM, secondary electron images of the Ni MPs (A, C) and Ni80 NPs (B, D) present as µm-sized agglomerates at dry conditions.

The results clearly show that the nano-sized particles (primary size: 80 nm) were not present as primary particles in solution but rather existed as micron-sized agglomerates. Agglomeration was also evident for the Ni MPs (Fig 4A). As will be discussed below from a theoretical perspective, this is an expected observation which can be explained by exceptionally strong attractive van der Waals forces between adjacent metallic particles both in air and solution due to large Hamaker constants (approximately 100 times larger for metals than for proteins and polymers, see Supporting information) [29]. Strong agglomeration of the nano-sized particles (Ni80 NPs) and the presence of some larger sized particles at dry conditions, were also observed by means of SEM imaging (Fig 4B).

Similar observations with Ni80 NPs present as large micron-sized agglomerates in solution were also observed in another cell medium (BEGM, see S1 Fig). These findings are also in concordance with a recent study using the same batch of Ni80 NPs reporting large micron-sized agglomerates present in both water and PBS [30].

**Fig 5:**
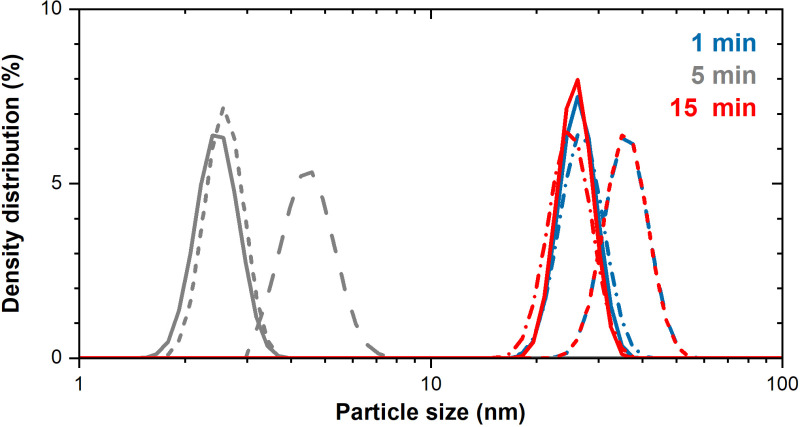
Effect of water bath sonication time on the size distribution of cell medium constituents. Changes in size distribution of cell medium (DMEM+) constituents (no metallic particles) sonicated using a water bath for 1, 5 or 15 min based on triplicate samples for each time period.

Even though considerable variations in particle size distribution in solution were observed across the quadruple measurements of both particle types for each sonication time (Fig 3), a clear trend emerged in both cases. The particle size distributions in solution shifted towards smaller sizes as the sonication time increased. This effect is induced by the delivered acoustic energy of the sonication bath, which enables partial deagglomeration and disruption of the µm-sized agglomerates into smaller, still mainly µm-sized agglomerates and possibly some smaller-sized particles into solution. The extent of disintegration would be expected to be more extensive if using tip sonication, which delivers a higher acoustic energy to the system than the water bath, as previously shown for e.g. NPs of Cu, Mn, ZnO and Al [7,9]. It should be emphasized that the delivered acoustic energy (increasing with time) also can influence the cell media constituents [4]. This is demonstrated by the observation that the size distribution of the cell medium constituents changed in a non-consistent way between the different sonication times (Fig 5), indicative of change in biomolecule conformation. Future studies by the authors will be conducted to assess the underlying reasons behind these changes. Similar findings were observed in BEGM cell medium (S2 Fig).

**Fig 6:**
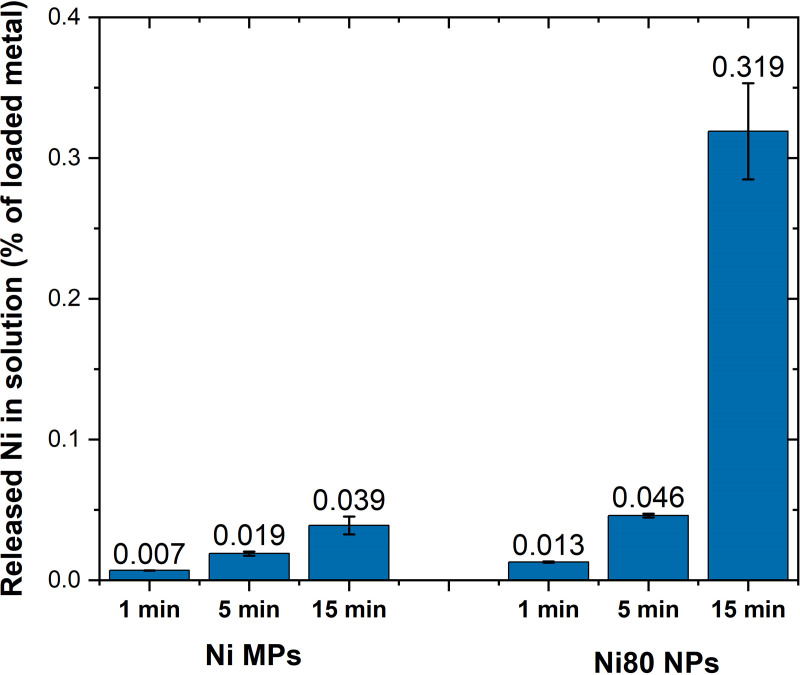
Effect of water bath sonication time on particle dissolution from Ni MPs and Ni NPs in cell medium. Released Ni fractions (% of the transferred Ni mass – mean value of three independent samples for each sonication time) of Ni MPs and Ni80 NPs in a stock solution of cell medium (DMEM+) measured directly after water bath sonication for 1, 5 or 15 min.

The increasing sonication time of the cell medium stock solution using a water bath did not only shift the particle size distribution of the Ni MPs and the Ni80 NPs to smaller sizes, Fig 3, it also influenced the extent of particle dissolution. This is illustrated in Fig 6, showing the extent of Ni dissolution determined by means of AAS immediately after 1, 5 or 15 min of water bath sonication of stock solutions of the nano- and micron sized Ni metal particles (Ni80 NPs and Ni MPs) in cell medium (DMEM+). Even though Ni was released to a very low extent, the effect was evident. The released Ni fraction increased with sonication time for both particle types. Less than 0.05% of the total particle mass was released from the Ni MPs into DMEM+ after 15 min of sonication, whereas the fraction for the Ni 80 NPs was 10 times higher (≈0.3%) after the same time. Less than 0.1% was released after 1 and 5 min of sonication. Similar trends were observed for the Ni80 NPs in BEGM cell medium (S3 Fig). Despite very small fractions of released Ni from both the micron- and the nano-sized particles, the results clearly show that an increased water bath sonication time results in an increased extent of Ni dissolution. The effect is most likely due to an increased particle surface area resulting from partial deagglomeration into smaller sized particles/agglomerates with increased sonication time (Figs 2 and 3). It could also be connected to changes in surface characteristics since sonication can result in water sonolysis forming reactive oxygen radicals which may oxidize the surfaces of metallic particles, and that the extent of oxidation is highly connected to the energy input and sonication time [4]. Another possible reason for increased particle dissolution with increasing sonication time could be related to an increase in temperature in the water bath unless circulating cooling systems are used. This further emphasizes that the sonication time needs to be as short as possible.

These findings show that the sonication process, depending on the sonication time, results in a stock solution that not only contains dispersed particles but also a fraction of released metals (as ions, labile or strong complexes) as well as partly dissolved particles/agglomerates. The extent of the dissolved fraction is sonication methodology-, solution-, and metal specific.

Previous findings by the authors and other studies have furthermore shown the choice of sonication method, i.e., the delivered acoustic energy, to influence the apparent surface charge of metallic particles, which is highly connected to the particle stability [9,31–33]. The effect of sonication on the zeta potential was not investigated in this study, however, the formation of smaller-sized particles/agglomerates with increased sonication time would most likely increase their zeta potential as previously shown for Al, Mn and ZnO NPs [9].

Overall, the results emphasize the need to optimize the time of the sonication process for a given particle type and exposure condition to minimize the extent of particle dissolution and possible changes in particle characteristics, while ensuring sufficient suspension stability of the stock solution. These aspects will influence the interpretation of results for a given investigated endpoint, for instance toxic effects induced by particles, dissolved species, or both.

### Effect of water bath sonication time on particle dispersions from a cytotoxicity perspective

**Fig 7:**
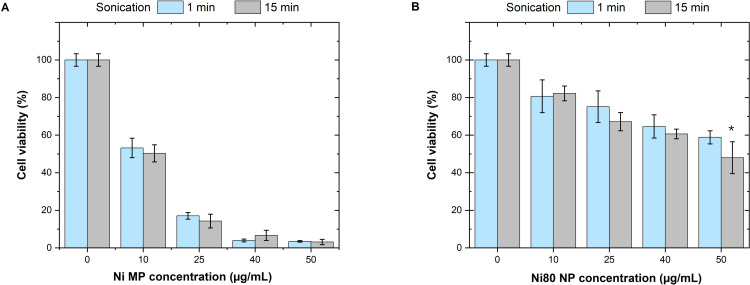
Effect of water bath sonication time on Ni MP and Ni80 NP particle cytotoxicity. Cytotoxicity of Ni MPs (**A**) and Ni80 NPs (**B**) assessed using the Alamar blue assay following exposure of A549 type II lung epithelial cells for 72 h after sonication (using a water bath) the stock solution for either 1 or 15 min. Two independent experiments were performed with similar results. The results are presented as mean ± SD of one experiment with five technical replicates. Asterisks indicate statistically significant differences (p < 0.05, two-way ANOVA followed by Šídák’s multiple comparisons test).

The effect of water bath sonication time (1 min or 15 min) of stock solutions of Ni MPs and Ni80 NPs in cell medium on the cytotoxic response following their exposure to A549 cells (type II lung epithelial cells) at submerged cell conditions were investigated using the Alamar blue assay. In the first set of experiments, a wide dose range was tested at exposure time 24 and 48 h, respectively, see S4 Fig. The results show no clear difference in cytotoxic response depending on the sonication time except for a higher toxicity observed after 48 h at a particle dose of 50 µg/mL for the Ni MPs (possibly also for the Ni80 NPs) sonicated for 15 min compared to 1 min. Since these effects could possibly be more pronounced after longer exposure periods, additional experiments were conducted for selected concentrations at 72 h exposure. In line with previous experiments, a slight increase in cytotoxicity was observed following 15 min of sonication, which in this case was significant for Ni80 (see Fig 7). Overall, it appears that sonication time may affect cytotoxicity, but the effect was relatively small.

Even though only small effects on cytotoxicity were observed in this limited study using water bath sonication of Ni NP and Ni MP stock solutions, previous findings by the authors clearly show that tip sonication of the stock solution of Cu NPs prior to cell exposures can result in both an increased toxic response (cell viability) and increased particle dissolution (release of Cu). Different particle concentrations exposed to A549 cells grown in DMEM cell medium with and without serum were investigated [8,34]. The underlying reasons for the observed increased toxicity were attributed to the formation of smaller-sized particles/ agglomerates, a large extent of particle dissolution in the stock solution (i.e., both particles and released ions/complexes), and changes in surface reactivity induced by the sonication process.

Overall, the results illustrate that the sonication step of the stock solution by different means may influence the toxic response and the extent of particle dissolution, though the effect is highly particle-, solution-, and sonication methodology (energy and time) dependent.

### Effect of sonication time on the transferred particle dose (concentration) and the importance of measuring dose samples

**Fig 8:**
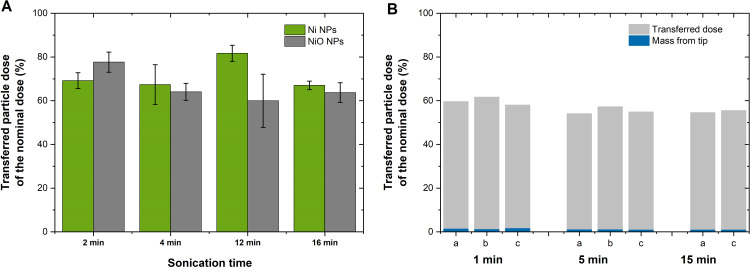
Effect of sonication time on the transferred dose compared with the nominal dose of Ni and NiO NPs in ultrapure water and cell medium. Transferred dose (% of the nominal dose) of Ni and NiO NPs (from Sigma; 100 nm) in ultrapure water stock solutions tip sonicated for 2, 4, 12 or 16 min (A), and for a water bath sonicated (1, 5 or 15 min) stock solution of Ni80 NPs (three replicate samples for each sonication time, except for 15 min sonication) in cell medium (DMEM+). The mass from the tip reflects a remaining mass of Ni in the pipette tip after pipetting (B).

Dose samples for each time point and particle type for the same measurements investigating the effect of tip sonication on the particle size and dissolution of Ni and NiO NPs (from Sigma, 100 nm) in ultrapure water (Fig 2) were prepared and compared to the nominal dose. The results are presented in Fig 8A showing approximately 20–40% lower transferred dose than the nominal dose for all time points. No conclusive effects with increasing tip sonication time could be discerned due to large variability between the triplicate samples for some of the time points. This implies non-homogeneous dispersions elucidating the necessity to prepare and analyze dose samples for each time-period and exposure conditions to assess the transferred doses. Dose samples should be prepared and analyzed for both nano- and micron-sized particles.

Dose samples were also prepared for Ni 80 NPs stock solutions in cell medium (DMEM+) water bath sonicated for 1, 5 or 15 min. The results are illustrated in Fig 8B showing considerably lower transferred doses than the nominal dose, being approximately 55–62% of the nominal dose, and some small variability within the three (two after 15 min) replicate samples for each sonication time. No conclusive trend in decreasing doses with increasing sonication time of the stock solution could be discerned even though a small but significant (p < 0.05) difference was calculated using the student t-test between 1 min of sonication and the longer sonication times.

**Fig 9:**
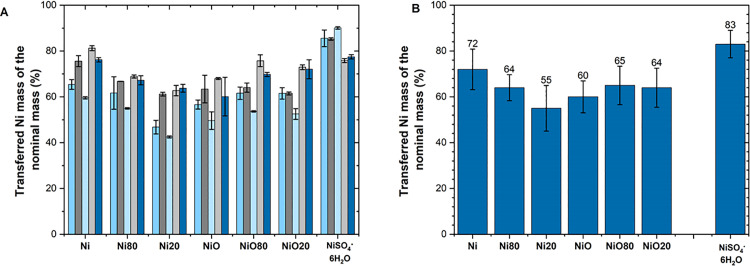
Transferred dose compared to the nominal particle dose of nano- and micron-sized Ni-based particles. Average transferred dose (% of the nominal (supposed) dose) based on triplicate readings of 5 unique dose samples each (A), and average doses of the five measurements (B) of Ni/NiO nano- and micron-sized particles transferred from a stock solution of BEGM cell medium (after 1 min water bath sonication) into the intended exposure vessels.

It should be emphasized that lower transferred doses than the nominal doses are not only an issue for nano-sized particles, but also micron also-sized particles show the same results because of strong van der Waal forces that govern their aggregation/settling patterns (see theoretical discussion below). This is illustrated in Fig 9A for stock solutions of Ni and NiO NPs of different primary size (20 and 80 nm; Ni20, Ni80, NiO20 and NiO80 NPs) and micron-sized particles (Ni and NiO MPs) as well as a nickel salt (NiSO_4_^.^6H_2_O) dispersed in cell medium (BEGM). The average results, Fig 9B, based on triplicate readings each of 5 independently prepared dose samples for each particle type, show that the transferred dose (based on Ni content) after 1 min sonication (water bath sonication) of the stock solution (BEGM cell medium) was largely varying between 55 and 72% of the nominal dose. The NiSO_4_ ∙ 6H_2_O particles showed a higher transferred amount (80–90%) than the other particles due to its high solubility in solution as a salt, i.e., less particle agglomeration, complexation and particle settling.

The results show the importance of preparing and assessing parallel dose samples for each exposure condition and type of metallic particle to determine the actual transferred dose of investigated particles and not rely on the nominal dose when determining the extent of particle dissolution or for dose-response toxicity studies as it shifts the released levels and the actual dose at which effects are seen. This has implications for regulatory threshold values for released metals and for dose-response toxicity effects induced by metallic particles on which government regulatory decisions and human exposure limits are based.

The results are in line with previous findings by the authors showing lower transferred particle doses of metal NPs than the nominal doses being on average approximately 60% for Cu NPs, 30–40% for Mn NPs, 70–80% for ZnO NPs and 40–60% for Al NPs in NaClO_4_ stock solutions [9]. The effect of sonication on the transferred dose has also been reported for particle dispersions of stock solutions of CuO, MgO and ZnO comparing sonication bath- and homogenizer treatments[10]. The results showed both higher (CuO and ZnO) and lower (MgO) transferred doses depending on the sonication treatment. Reduced transferred doses with increasing sonication time has also been observed for ZnO NPs in NaClO_4_ [9].

In all, considerably lower transferred particle doses compared with the nominal doses were observed for both micron- and nano-sized Ni-based particles being either tip-sonicated in ultrapure water, or water bath sonicated in cell medium. No evident effect of sonication time was observed. Lower transferred doses are related to losses during transfer of solution from the stock solution, effects of particle agglomeration and settling (sedimentation), as well as remnants on the tip and on the tube walls of the transferring pipette. The results clearly illustrate the necessity to determine the transferred dose for each exposure condition and particle type (both nano- and micron-sized particles) when preparing particle dispersions for, e.g., *in vitro* and *in vivo* toxicity testing.

### Theoretical considerations – effects of strong van der Waals forces on Ni and NiO particles from a particle agglomeration and settling perspective

Non-functionalized metallic particles (both nano- and micron-sized) have a general tendency to form agglomerates in solution because of a combination of intrinsic attractive forces, high surface energy, and environmental conditions that favor aggregation/agglomeration. Their high surface energy makes them energetically unfavorable in their dispersed state, a state which becomes minimized due to particle agglomeration taking place because of exceptionally strong attractive van der Waals forces, both in air and solution. The magnitude of this force depends on particle size and material properties, such as the dielectric constant and refractive index, represented by the Hamaker constant. Smaller sized particles have been shown to have higher Hamaker constants than larger sized particles [35]. The Hamaker constants for metals are significantly higher than for other materials. Therefore, metal particles without any surface modification (both MPs and NPs) show a strong tendency to agglomerate and settle [29]. The extent of agglomeration depends also on other characteristics such as the surface charge which influence the repulsive electrostatic double layer force (see S1 Appendix), which is dependent on the solution chemistry (pH, ionic strength) [36]. These aspects are further explained in Supporting information (see S1 Appendix).

**Fig 10:**
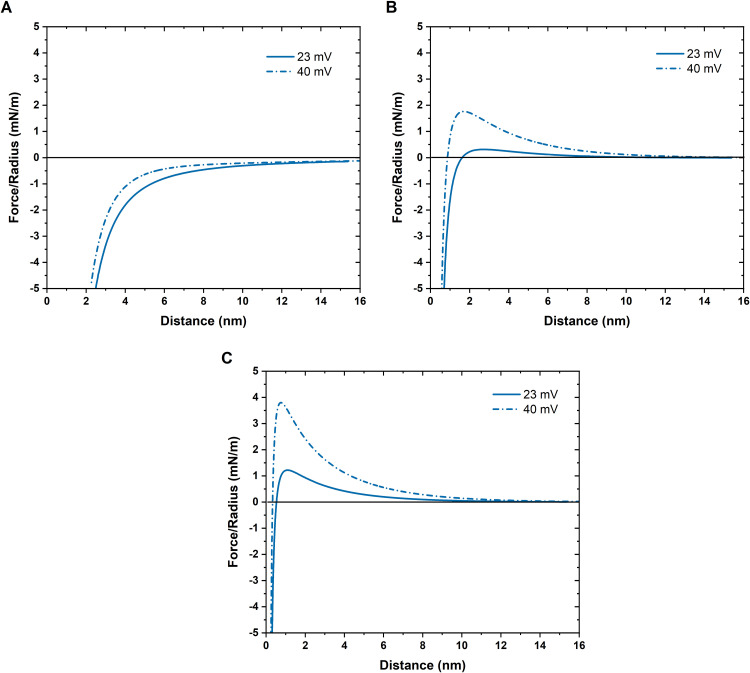
Calculated forces between Ni and NiO particles with and without a biocorona. Calculated DLVO forces normalized by the particle radius as a function of surface separation for Ni (A), NiO (B) and biomolecule-coated (C) particles using Hamaker constants (see Supporting information) for two different surface potentials (Zeta potentials), ± 23 mV and ± 40 mV.

The DLVO (Derjaguin, Landau, Verwey, and Overbeek) theory, which considers attractive van der Waals forces and repulsive electrostatic double-layer forces between particles, was employed to theoretically estimate the stability of Ni and NiO particles (independent of size since planar surfaces are assumed) in solution and assess effects of an adsorbed layer of, e.g., biomolecules, a biocorona [37–39]. The van der Waals forces were calculated for Ni and NiO particles and for biomolecules using Hamaker constants based on assumptions and literature findings (see Supporting information). The calculated forces between two particles, normalized by the particle radius as a function of surface separation, is presented in Fig 10 for particles of Ni (A), NiO (B) and biomolecules (C) at two different apparent surface potentials, Zeta potentials (23 and 40 mV). The results show that the magnitude of the van der Waals attraction dominates over the electrostatic repulsion of the interaction at all surface separations (Fig 10A) and for both surface potentials. This leads to rapid particle agglomeration to an extent which also can result in settling of formed agglomerates. For the NiO particles (Fig 10B) a higher repulsive barrier was determined for a potential of 40 mV compared with 23 mV, which may induce some stabilization of NiO particles in a 10 mM monovalent electrolyte. However, even though surface charge measurements (zeta-potential) would imply particle stability in solution (typically ±30 mV), extensive agglomeration may still be taking place for metallic particles [40]. This is in qualitative agreement with the particle stability measurements of Ni and NiO NPs in Fig 2, showing higher particle concentrations in ultrapure water of the NiO NPs compared to the Ni NPs even though other factors like density, agglomeration and dissolution also play a role. In the case of biomolecule-coated surfaces/particles (e.g., in the presence of a protein corona) (Fig 10C) a repulsive barrier is present for both potentials. The van der Waals attraction exceeds the repulsion at a short range (closer to the surface), i.e., the particles become more stable in solution. Corresponding DLVO calculations assessing the role of a surface oxide on metal Ni particles clearly elucidate that the presence of a surface oxide or a biocorona must be sufficiently thick (>9 nm) for the electrostatic forces to play a role (S1 Appendix).

Detailed theoretical explanations on the influence of ionic strength, presence of surface oxide and biomolecule surface interactions are given in Supporting information together with connected aspects on zeta potential measurements.

In all, the intrinsic strong van der Waal forces between metallic particles in aqueous solution mainly governs their rapid and extensive particle agglomeration pattern both at dry conditions and in solutions containing biomolecules (in this study cell medium constituents) forming a biocorona. From this follows an evident higher tendency for rapid particle/agglomerate settling even after solution sonication of non-coated nano- and micron-sized Ni and NiO particle dispersions for *in vitro* and *in vivo* testing. The sonication step hence needs to be as short as possible, minimizing effects on particle size distributions, agglomeration/settling and dissolution.

### Concluding remarks

The results generated in this paper demonstrate that an increased sonication time not only reduces the particle size in solution (i.e., increases the exposed surface area), but it also increases the dissolved Ni fraction. More manipulation of the particles to weaken the strong van der Waal forces (longer sonication time, i.e., increased energy) will not only induce more Ni release but also largely change the particle size and the surface characteristics. Such effects should be minimized to ensure testing that is more representative of actual human exposure to the particles of interest and not a mixture of particles and dissolved metals (here Ni), induced by the sonication process, already in the stock solution (time zero). Although sonication or other types of high energy input dispersion techniques are commonly used in toxicological studies, these results emphasize the need to determine the extent of sonication that is most appropriate for the purpose, study design, and test particle, without significantly impacting particle surface characteristics, particle size, particle dissolution, and possibly toxicity.

While similar findings have been reported in the literature, a deeper investigation is warranted. The authors are conducting follow-up studies using different cell culture media and another cell line to enable further comparison and exploration. The results further emphasize the need to reassess the extent of sonication, striking a balance between effective particle dispersion and minimal impact on toxicity outcomes (e.g., bioaccessibility, cytotoxicity), and the importance of considering the relevance of sonication to the specific study type. Intrinsic particle properties, study type, and the degree of sonication are critical factors to consider when designing particle dispersion protocols in research.

In all, sonication can potentially have a huge impact on study results due to effects on particle characteristics, particle size, and particle dissolution. Generated results may hence completely differ depending on the choice and operational settings of the sonication protocol. Additionally, an accurate indication of the actual transferred dose compared to the nominal dose is important, as it can significantly differ. Determining the actual transferred dose does not change or impact the end results, however, it does shift the dose at which effects are observed. Both the extent of sonication and the determination of transferred dose are hence particularly critical for toxicity studies on which government regulatory decisions and human exposure limits are based.

## Supporting information

S1 Fig Changes in hydrodynamic particle size distributions of the nano-sized (Ni80 NPs) particles in cell medium (BEGM) ultrasonicated for 1, 5 or 15 min in a water bath. Data is presented as the density distribution by volume with corresponding scattered light intensity (B). Four independent samples were investigated for each sonication time point.
(PDF)

S2 Fig Changes in size distribution of cell media (BEGM) constituents ultrasonicated using a water bath for 1, 5 or 15 min. (PDF)

S3 Fig Released Ni fraction (% of the administrated Ni mass – mean value of three independent samples for each sonication time) of Ni80 NPs in a stock solution of cell medium (BEGM) ultrasonicated in a water bath for 1, 5 or 15 min prior to analysis. (PDF)

S1 Table Particle size distribution of Ni and NiO NPs in ultrapure water (data from Fig. 2a). (PDF)

S4 Fig Effect of water bath sonication time on Ni MP and Ni80 NP particle cytotoxicity. Cytotoxicity of Ni MPs (A) and Ni80 NPs (B) assessed using the Alamar blue assay following exposure of A549 type II lung epithelial cells for 24 and 48 h after sonication (using a water bath) the stock solution for either 1 or 15 minutes. The results are presented as mean ± SD of two independent experiments, each with triplicates. PC. = positive control. Asterisks indicate statistically significant differences (***adj. p ≤ 0.001, two-way ANOVA followed by Šídák’s multiple comparisons test).
(PDF)

S1 Appendix Particle stability – theoretical background and calculations. (PDF)
