# Supplementary material for: Effects of sonication on particle dispersions from a size, biodissolution, cytotoxicity and transferred dose perspective – a case study on nickel and nickel oxide particles
Source: PLoS One. 2025 May 9;20(5):e0323368. doi: 10.1371/journal.pone.0323368 (PMC12063897; doi:10.1371/journal.pone.0323368)
Supplement: S1 Fig — Data is presented as the density distribution by volume with corresponding scattered light intensity (B). Four independent samples were investigated for each sonication time point. (PDF) [file pone.0323368.s001.pdf]

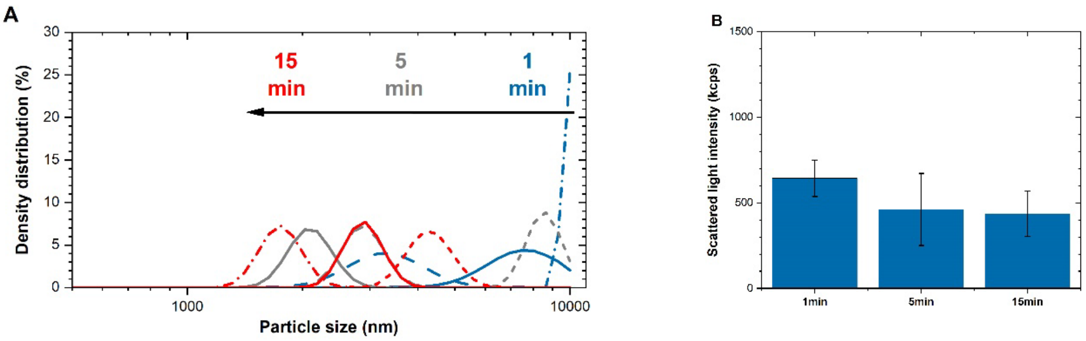

***S1 Fig. Changes in hydrodynamic particle size distributions of the nano-sized (Ni80 NPs) particles in cell medium** (BEGM) ultrasonicated for 1, 5 or 15 min in a water bath. Data is presented as the density distribution by volume with corresponding scattered light intensity (B). Four independent samples were investigated for each sonication time point.*
