# Supplementary material for: Effects of sonication on particle dispersions from a size, biodissolution, cytotoxicity and transferred dose perspective – a case study on nickel and nickel oxide particles
Source: PLoS One. 2025 May 9;20(5):e0323368. doi: 10.1371/journal.pone.0323368 (PMC12063897; doi:10.1371/journal.pone.0323368)
Supplement: S2 Fig — (PDF) [file pone.0323368.s002.pdf]

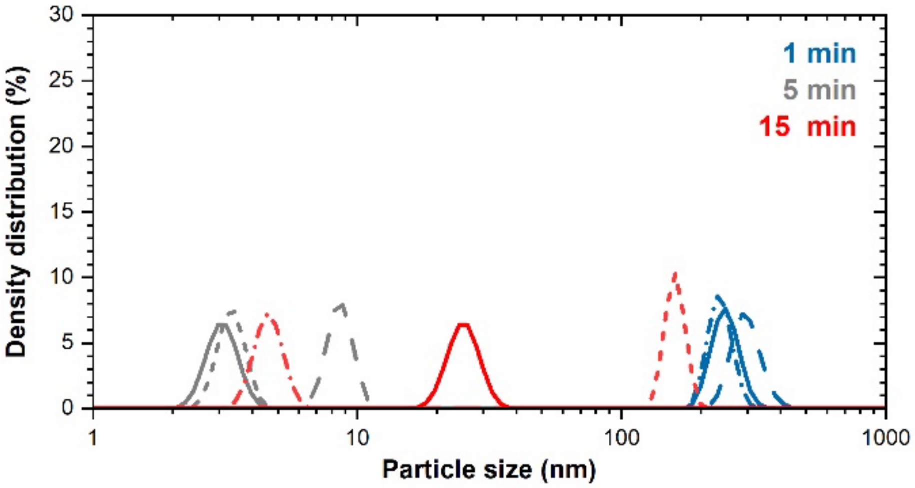

S2 Fig. Changes in size distribution of cell media (BEGM) constituents *ultrasonicated using a water bath for 1, 5 or 15 min (triplicate samples for each sonication time).*
