# Supplementary material for: Effects of sonication on particle dispersions from a size, biodissolution, cytotoxicity and transferred dose perspective – a case study on nickel and nickel oxide particles
Source: PLoS One. 2025 May 9;20(5):e0323368. doi: 10.1371/journal.pone.0323368 (PMC12063897; doi:10.1371/journal.pone.0323368)
Supplement: S1 Table — (PDF) [file pone.0323368.s004.pdf]

S1 Table. Particle size distribution of Ni and NiO NPs in ultrapure water (*data from Fig. 2a).*

	NI NPS		NIO NPS	
**SONICATION TIME**	**Mean size (nm)**	**STDAV**	**Mean size (nm)**	**STDAV**
**2**	136.6	7.33	136.0	7.65
**4**	132.8	5.08	132.3	3.38
**6**	136.9	7.89	133.6	3.25
**8**	127.7	5.97	130.2	3.21
**10**	130.5	2.42	132.7	4.74
**12**	132.0	3.14	131.9	4.03
**14**	120.9	13.7	128.8	1.33
**16**	120.0	1.45	129.0	3.53
