# Supplementary material for: Effects of sonication on particle dispersions from a size, biodissolution, cytotoxicity and transferred dose perspective – a case study on nickel and nickel oxide particles
Source: PLoS One. 2025 May 9;20(5):e0323368. doi: 10.1371/journal.pone.0323368 (PMC12063897; doi:10.1371/journal.pone.0323368)
Supplement: S4 Fig — Cytotoxicity of Ni MPs (A) and Ni80 NPs (B) assessed using the Alamar blue assay following exposure of A549 type II lung epithelial cells for 24 and 48 h after sonication (using a water bath) the stock solution for either 1 or 15 minutes. The results are presented as mean ± SD of two independent experiments, each with triplicates. PC. = positive control. Asterisks indicate statistically significant differences (***adj. p ≤ 0.001, two-way ANOVA followed by Šídák’s multiple comparisons test). (PDF) [file pone.0323368.s005.pdf]

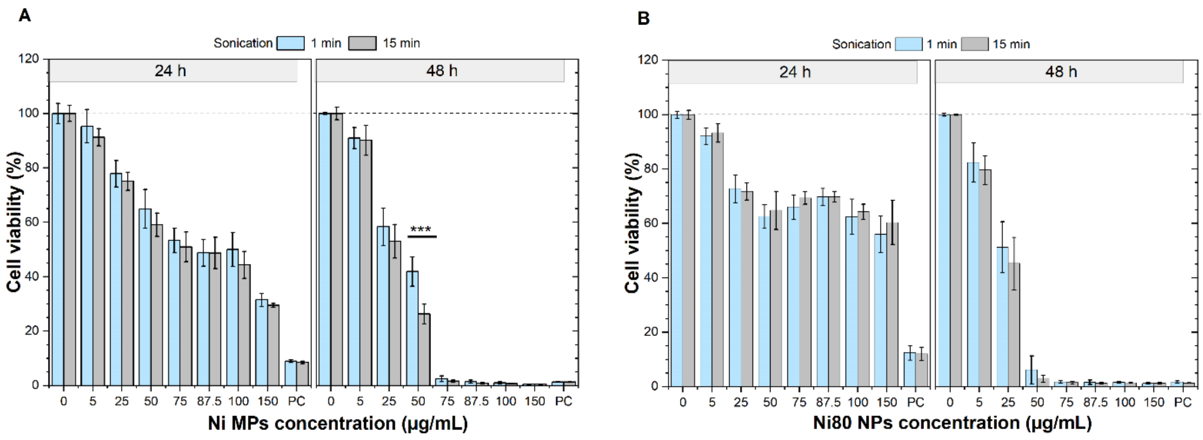

S4 Fig. Effect of water bath sonication time on Ni MP and Ni80 NP particle cytotoxicity.
*Cytotoxicity of Ni MPs (A) and Ni80 NPs (B) assessed using the Alamar blue assay following exposure of A549 type II lung epithelial cells for 24 and 48 h after sonication (using a water bath) the stock solution for either 1 or 15 minutes. The results are presented as mean ± SD of two independent experiments, each with triplicates. PC. = positive control. Asterisks indicate statistically significant differences (***adj. p≤0.001, two-way ANOVA followed by Šídák's multiple comparisons test).*
