# Supplementary material for: Effects of sonication on particle dispersions from a size, biodissolution, cytotoxicity and transferred dose perspective – a case study on nickel and nickel oxide particles
Source: PLoS One. 2025 May 9;20(5):e0323368. doi: 10.1371/journal.pone.0323368 (PMC12063897; doi:10.1371/journal.pone.0323368)
Supplement: S1 Appendix — (PDF) [file pone.0323368.s006.pdf]

PARTICLE STABILITY – THEORETICAL BACKGROUND AND CALCULATIONS

Metal (nano)particles undergo various transformation/dissolution processes, which are dependent on the surface oxide characteristics and the chemical environment.

Upon exposure to air, most pristine metal particles (MePs) immediately form a surface oxide layer (core/shell particles), as depicted in S4 Fig. The properties and composition of this surface oxide, along with the solution chemistry, determine its effectiveness in mitigating corrosion, which varies based on the type of metal. While a surface oxide can effectively mitigate corrosion of highly corrosion-resistant metals and alloys, metals can still dissolve (be released) from the material into solution. This takes place to varying degrees due to different electrochemical and chemical processes (such as proton-induced dissolution, complexation/ligand-induced dissolution, and reductive dissolution), which also alter the characteristics of the surface oxide. These highly dynamic reactions depend largely on the type of metal and the solution chemistry.

Once released, metals may exist as free or labile ions in solution, form strong metal complexes, or even lead to the formation of secondary metal nanoparticles (colloids) through complexation and precipitation processes with chemical components in the aqueous solution. Interactions with the chemical constituents of the solution can further influence surface interactions, where ligands adsorb or desorb to/from the particle surface, forming a biocorona. This biocorona affects particle properties such as charge, reactivity, composition, stability, and mobility.

Understanding changes in both the physicochemical properties of the MePs and the effects of interactions with chemical constituents in a test fluid, such as a biologically relevant medium, is hence critical since the surface of the particles serves as the interface with the environment which largely governs the particle behavior. These concepts are schematically illustrated in S5 Fig.

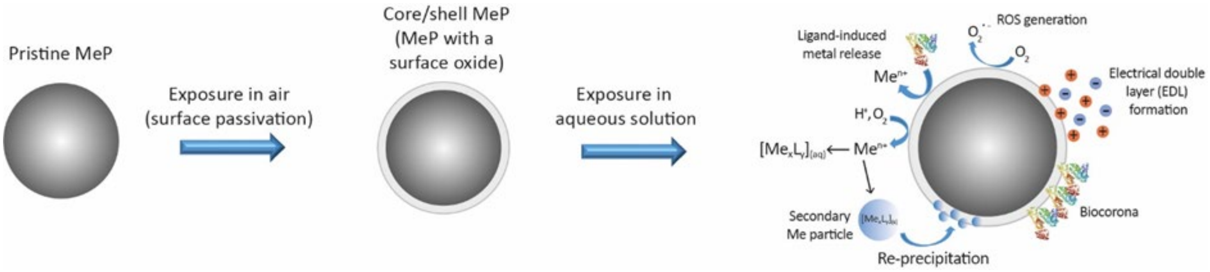

S5 Fig. Chemical transformations of pristine MeP in air and aqueous solution involve several processes. *The MeP undergoes surface passivation in air, forming a surface oxide. In solution, the MeP undergoes oxidative dissolution (Men+), changes in surface charge, biomolecule adsorption, and interactions with small molecules, resulting in modified MeNPs. Released metal ions interact with inorganic ligands and biological components to form soluble (MexLy) or insoluble (MexLy) complexes. These precipitates can result in secondary MePs, which, e.g. can re-precipitate onto the MeP surface. Images drawn using Adobe Illustrator.*

The dissolution of MePs follows the Gibbs–Thomson effect, which predicts that MePs with a smaller radius of curvature are energetically unfavorable and more prone to dissolution, leading to higher equilibrium solubility compared to micron-sized particles [1]. As previously mentioned, the driving force for dissolution largely also depends on the solubility of the surface oxides of the MePs in a given fluid, the concentration gradient between the particle surface and the bulk

solution phase, the stability and aggregation states of the MePs, their functionalization, and the ionic strength, pH, and nature of adsorbing species of the solution [2, 3].

Metal particles spontaneously gain an electrical surface charge in polar solutions, such as water.

When a metal oxide surface interacts with water molecules, it initially undergoes hydration, with water molecules being adsorbed. Subsequently, it undergoes hydroxylation, where hydrogen from the adsorbed water molecule migrates to the oxygen atom of the oxide [4].

When MePs are dispersed in an aqueous medium, they become solvated. The thickness and nature of the solvated layer is affected by solution conditions such as pH, ionic strength, temperature, and pressure [4, 5]. Interactions between the charged metal surface and the aqueous medium lead to the formation of an electrical double layer (EDL), which consists of an inner layer closest to the surface, which is considered immobile, potentially containing adsorbed ions (often denoted the Stern layer), and a diffuse outer layer, which lies further from the surface and permits the diffusion of ions, Fig S6A. The distribution of these ions in the layer is influenced by both electrical forces and random thermal motion. The dynamic structure of the EDL influences the charge distribution, particle stability and the electrochemical behavior of metallic particles in solution.

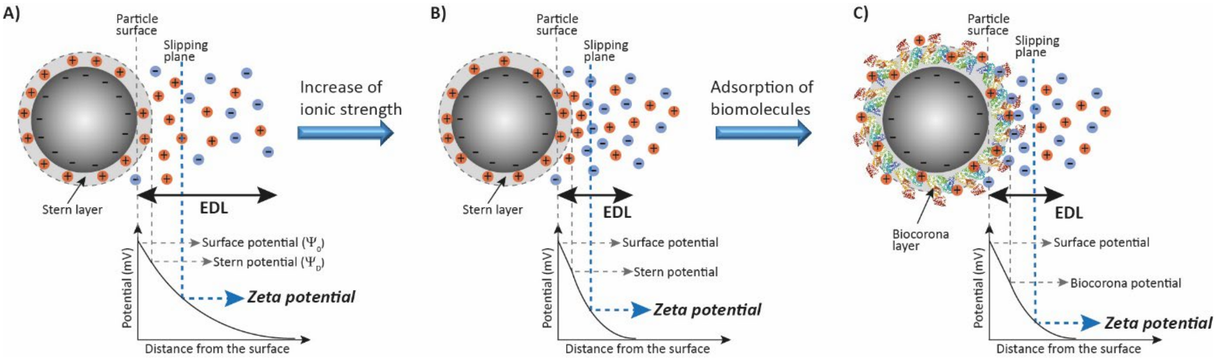

S6 Fig. ***Illustration depicting the variation in ionic concentration and potential difference with the distance from the charged surface of a particle suspended in an aqueous medium.*** *Images drawn using Adobe Illustrator.*

The electric potential decreases linearly from the actual thermodynamic surface potential, Ψ_0, to the Stern potential, Ψ_D, and then exponentially decays to zero within the outer diffuse layer, Figure S6A. This relationship is described by the Debye-Hückel equation (Eq. 1) [6]:

$$\Psi_0 = \Psi_D \exp(-\kappa \cdot x) \qquad (1)$$

where x represents the distance from the particle surface, and κ, known as the Debye-Hückel parameter, is defined by (Eq. 2) [6, 7]:

$$\kappa = \left(\frac{2e^2 N_A c z^2}{\varepsilon_r \varepsilon_0 k_b T}\right)^{1/2} \qquad (2)$$

In this equation, e denotes the protonic charge, N_A stands for Avogadro's constant, c denotes the concentration of electrolyte of valence z, ε_r represents the dielectric constant of the solution, ε_0

signifies the permittivity of vacuum, k_b is the Boltzmann constant, and T is the thermodynamic temperature. This expression is valid for a $z - z$ electrolyte, i.e. a symmetrical, binary electrolyte where the cation valence is z and the anion valence is $-z$ [6, 8].

The electric potential, hence, via κ, relies on the ionic composition of the medium. An increase in κ (e.g., an increase of the ionic strength) leads to the compression of the EDL, which results in a reduced potential, S6B Fig. Moreover, a higher valency of the ions in the solution will compress the EDL to a larger extent compared to monovalent ions [6, 9].

Adsorption of biomolecules on the MePs, forming a biocorona may further yield a lower electric potential because the slipping plane becomes positioned further away from the Stern plane, S6C Fig [10-12]. Since the biocorona also will shield the potential of the MePs, the measured potential will rather mainly equal the biocorona rather than the MePs [13, 14]. The adsorbed biomolecules in the biocorona may furthermore have an opposite charge compared to the metal surface, and, hence, an opposite potential may be measured.

An important aspect to remember is that the electric potential cannot be measured at the actual particle surface but rather at the slipping plane, denoted the Zeta potential (S6 Fig), at varying distances from the surface depending on the chemical solution conditions. The Zeta potential is, hence, always smaller than the actual surface potential of the metal.

The surface charge can indirectly be determined via Zeta potential measurements, though it is highly solution dependent.

The surface charge can indirectly be determined by means of Zeta potential measurements, commonly using electrophoretic light scattering (ELS). Electrophoresis refers to the motion of charged colloidal particles within a medium when subjected to an external electric field. The applied electrical field causes the charged particles to move, S7 Fig, and the applied voltage enables the dispersed particles to be drawn towards the electrode of opposite polarity. This particle movement involves the fixed layer and a portion of the diffuse double layer, specifically the internal side of the "sliding surface". As the MePs move within the aqueous medium, a boundary emerges between the ions in the diffuse layer that move along with the particles and the ions that remain within the bulk phase. The electrostatic potential at this boundary, known as the "slipping plane", is denoted by the Zeta potential; see S7 Fig.

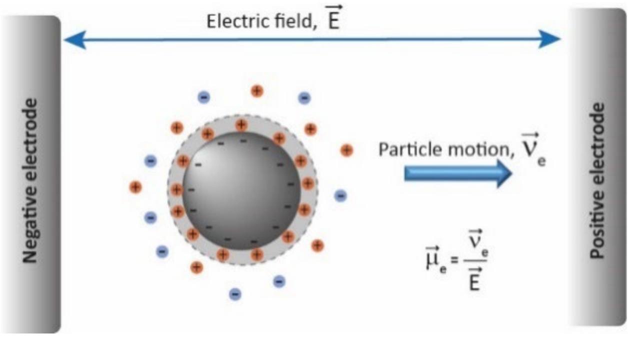

S6 Fig. Schematic showing the mechanism of Zeta potential measurements using electrophoresis. *Image drawn using Adobe Illustrator.*

The electrophoretic mobility (μ_e, $m^2V^{-1}s^{-1}$), defined as the ratio between the electrophoretic velocity (v_e, ms^{-1}) of the MePs and the external applied electric field strength (E, V), can be measured and converted to the Zeta potential (ζ) by applying the Henry equation (Eq. 3) [7, 8]:

$$\mu_e = \frac{2 \cdot \varepsilon_r \cdot \varepsilon_0 \cdot \zeta \cdot f(\kappa \cdot \alpha)}{3 \cdot \eta} \tag{3}$$

where ε_r and η represent the dielectric constant and the absolute zero-shear viscosity of the medium, respectively. The function $f(\kappa \cdot \alpha)$ is referred to as the "Henry function", where α represents the particle radius, and κ is denoted as the Debye parameter, signifying the thickness of the EDL that depends on the ionic strength of the medium and the temperature of the sample, being $1/\kappa$ (discussed in the next section). The Henry equation is often accompanied by several potential approximations, typically aligning with either the Smoluchowski or Hückel approaches, where $f(\kappa \cdot \alpha)$ is presumed to be either 1 (Hückel) or 1.5 (Smoluchowski) [15]. The primary disparity between these approximations lies in their assumptions regarding the thickness of the EDL compared to the particles themselves. The Smoluchowski approximation suggests that the EDL is considerably thinner than the particle radius, whereas the Hückel approximation posits the double layer to be significantly thicker than the radius of the particles [5, 9, 15-19]. The Smoluchowski approximation is given by (Eq. 4) [15]:

$$\mu_e = \frac{\varepsilon_r \cdot \varepsilon_0 \cdot \zeta}{\eta} \tag{4}$$

It should be emphasized that Smoluchowski's approximation is valid for particles of any shape as long as the radius of curvature at every point on the surface is significantly larger than the EDL thickness ($1/\kappa$) and, thus, that the particle surface can be considered to be locally planar [7, 20].

Higher charged particles will move faster in the electrical field. An increase in ionic strength (i.e. a lower $1/\kappa$) will result in more ions being available, and the initial surface charge can be compensated much faster, which results in lower electrophoretic mobility and, hence, a lower Zeta potential [8]. When measuring the Zeta potential using ELS, it is assumed that the samples are monodisperse and free from aggregates or agglomerates. For MePs, this is seldom the case. Larger-sized particles/aggregates can, in polydisperse samples, bias the mobility measurements since light scattering increases with particle size [19]. Hence, in addition to mean values, reporting of Zeta potentials should also include intensity distribution curves that justify the quality and reliability of the measurements. In biomolecule-containing cell media (such as DMEM or BEGM), signals from, for example, proteins in solution can interfere with the measured Zeta potential. Therefore, the intensity distribution curves for the blank solution without particles should always be reported for solutions containing biomolecules, e.g., cell media [13].

The magnitude of the measured Zeta potential reflects the extent of electrostatic repulsion between adjacent, similarly charged particles in dispersion. For molecules and particles that are small enough, a high Zeta potential will confer particle stability, i.e., limited agglomeration. At low Zeta potentials, the attractive forces may exceed this repulsion, and agglomeration will take place. Particles that show high Zeta potential (negative or positive) are electrically stabilized while agglomerating if having low Zeta potentials. Particles with Zeta potentials more positive than +30 mV or more negative than -30 mV are normally considered stable in solution [14].

Metal (nano)particles readily aggregate at both dry and wet conditions due to large van der Waals forces

Overall, the tendency for non-stabilized MePs to agglomerate in solution is a result of a combination of intrinsic attractive forces, high surface energy, and environmental conditions that favor aggregation/agglomeration. Their high surface energy makes them energetically unfavorable in their dispersed state. This state becomes minimized via particle aggregation taking place as a consequence of exceptionally strong attractive van der Waals forces (vdW) between metal particles (both in air and solution), forming larger aggregates than the pristine particles with presumed lower overall surface areas [21].

The vdW force is always attractive between similar particles in any medium, resulting from interactions between induced and/or permanent dipoles. The magnitude of the vdW force depends on particle size and material properties, such as the dielectric constant and refractive index, represented by the Hamaker constant. At large particle separations, the vdW interaction is primarily influenced by the properties of the bulk material, whereas at short separations, it is dominated by the surface layer (e.g., surface oxide) characteristics [22]. The Hamaker constants for metals are significantly higher than for other materials since they are highly conductive and polarizable, i.e. they have high dielectric constants and refractive indices. Therefore, metal MPs and NPs without any surface modifications exhibit a strong tendency to aggregate and precipitate (settle) in aqueous solutions due to the strong vdW forces [23].

The vdW force between two spheres of constant radii (R_1 and R_2) as a function of separation (D) is given by (Eq. 5) [22]:

$$F = \frac{-A}{6 \cdot D^2}\left(\frac{R_1 \cdot R_2}{R_1 + R_2}\right) \quad (5)$$

The non-retarded vdW force between two macroscopic particles can be determined using the Hamaker constant, A, which depends on the chemical properties of the particles and applies to any macroscopic geometry. Typically calculated using Lifshitz theory (Eq. 6), the Hamaker constant indicates the strength of vdW forces with higher values corresponding to stronger attractive forces (Eq. 6) [22, 24]. For conducting materials like metals with high dielectric properties and refractive indices, the Hamaker constant is significantly higher than for non-conducting materials (Eq. 6), leading to strong vdW forces and a higher tendency for agglomeration. The Hamaker constant for two macroscopic bodies 1 and 2 in a medium 3 can be calculated according to the Lifshitz theory as follows (Eq. 6)[22, 24]:

$$A = \frac{3}{4}kT\left(\frac{\varepsilon_1-\varepsilon_3}{\varepsilon_1+\varepsilon_3}\right)\left(\frac{\varepsilon_2-\varepsilon_3}{\varepsilon_2+\varepsilon_3}\right) + \frac{3h\upsilon_e}{8\sqrt{2}}\frac{(n_1^2-n_3^2)(n_2^2-n_3^2)}{(n_1^2+n_3^2)^{1/2}(n_2^2+n_3^2)^{1/2}\left[(n_1^2+n_3^2)^{1/2}+(n_2^2+n_3^2)^{1/2}\right]} \quad (6)$$

where ε is the static dielectric constant, υ_e is the main electronic absorption frequency in the UV region (often about $3 \cdot 10^{15}$ Hz, assumed to be the same for all three media, h is the Planck's constant, n is the refractive index in the visible region, k is the Boltzmann's constant, and T is the absolute temperature.

From Eq. 6, it is evident that the vdW force between two identical particles in a medium is always attractive (A positive), while it can be attractive or repulsive (A negative) between different particles in a medium. Moreover, the vdW force is always attractive in air or vacuum, and the magnitude of the vdW force decreases in an aqueous solution.

As a result of strong vdW forces, extensive aggregation in solution will take place for both nano- and micron-size MePs. These aggregates, depending on size and density, readily settle (sediment) from the solution with time. However, the extent of aggregation is also dependent on other characteristics such as the surface charge, which is dependent on the solution chemistry, and the formation of a biocorona as described above, see S4 Fig.

Aggregation/agglomeration of Ni and NiO particles in solution is governed by van der Waals attraction forces rather than by electrostatic repulsion

The DLVO (Derjaguin, Landau, Verwey, and Overbeek) theory was employed to theoretically estimate the stability of metallic Ni particles in solution and assess the role of particle size, surface oxide thickness and effect of an adsorbed layer of, e.g. biomolecules, a biocorona. The DLVO theory considers the attractive van der Waals (vdW) forces and the repulsive electrostatic double-layer (EDL) forces between particles. The extent of particle agglomeration is determined by the sum of these vdW and EDL forces, representing the total interaction force between colloidal particles [6, 8, 9].

Calculated DLVO forces normalized by the particle radius as a function of surface separation are presented in the main paper, Fig 10. The vdW forces were calculated for Ni and NiO (nano)particles using the Hamaker constant, based on literature findings in S2 Table, and the EDL force, using the nonlinear Poisson-Boltzmann approximation and assuming constant charge interaction, based on the algorithm of Chan et al. [25]. In monovalent electrolyte solutions, the decay length of the double-layer force is accurately described by the Debye length (κ^{-1}). These theoretical values were used in all calculations unless specified otherwise. It was assumed that the plane of charge and the origin of the vdW force are located at the surface.

***S2 Table. Hamaker constants**, based on literature findings, in an aqueous solution for Ni and NiO particles used in the DLVO calculations.*

Particle	Hamaker constant ($\cdot 10^{-20}$ J)
**Ni**	21.9[a]
**NiO**	2.19[b]
**Hydrocarbon/protein**	0.5[c]

[a] *From reference [26]*
[b] *Assumed to be ~1/10 of A for Ni metal [27]*
[c] *From reference [22]*

At the maximum of the energy barrier, repulsive forces exceed attractive forces. Consequently, particles rebound upon contact and remain dispersed in the medium. For this dispersion to be maintained, the energy barrier must exceed the thermal energy. If lower, the particles will aggregate due to attractive forces. The energy barrier height reflects the stability of the system, as particles need to overcome it to aggregate. For two colliding particles to aggregate, they must possess enough kinetic energy, determined by their velocity and mass. If they surpass the barrier, the net interaction becomes attractive, leading to aggregation. This barrier is often called an energy trap because the vdW forces effectively keep the particles together [22].

The DLVO theory primarily accounts for the EDL repulsion and the attractive vdW forces. However, other forces, such as steric repulsion, play a significant role, particularly when

biomolecules are adsorbed onto the metallic particles. Steric repulsion is a result of volume restrictions and interpenetration effects of the adsorbed molecules impacting particle agglomeration. When two particles with adsorbed biomolecules, such as proteins in a biocorona, approach each other, they can repel one another due to the overlapping or compression of the adsorbed layers. This steric repulsion adds to the overall repulsive forces between particles. In the same way, the attractive vdW force is reduced for particles coated with an organic layer, as the Hamaker constant for the organic layer is considerably lower compared to that of the metal NP, see S2 Table.

For surfaces coated with a thin layer of different dielectric properties than the metal surface (e.g. a surface oxide or an adsorbed protein layer (protein corona)), the vdW and the effective Hamaker constant can be calculated (Eq. 7) assuming a three-layer system consisting of the metal (1), a surface oxide layer/biocorona (2) and the solution (3) [22, 28].

$$\frac{F(D)}{R} = -\frac{1}{6}\left(\frac{A_{232}}{D^2} - \frac{2A_{123}}{(D+T)^2} + \frac{A_{121}}{(D+2T)^2}\right) = -\frac{A_{eff}(D)}{6D^2} \quad (7)$$

where A_{232} = the Hamaker constant for surface layer-solution-surface layer, A_{123} = the Hamaker constant for metal-surface layer-solution, A_{121} = the Hamaker constant for metal-surface layer-metal, A_{eff} = the effective Hamaker constant, D = the distance between particles, and T = the thickness of the surface layer.

S8 Fig illustrates how the effective Hamaker constant, calculated at a particle distance of 1 nm using Eq. 7, varies with the thickness of the surface layer. As seen in the figure for Ni particles coated with a surface oxide (Ni-NiO) or a protein corona (Ni-protein), a layer with a thickness larger than approximately 9 nm is required to reduce A_{eff} to a Hamaker constant with a value close to that of NiO or a protein (S2 Table). This will, in turn, lead to a lower vdW force and, hence, a DLVO interaction similar to that for NiO and hydrocarbon/protein, see Figs 10B and 10C in the main paper.

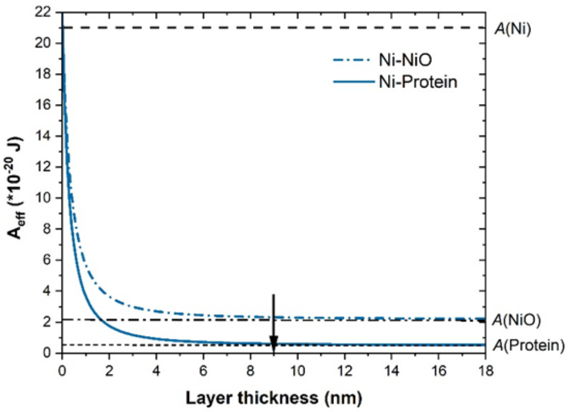

***S8 Fig. The calculated effective Hamaker constant (Aeff) for Ni metal particles as a function of the thickness of a surface layer** (surface oxide: Ni-NiO, or biocorona: Ni-protein) is calculated at a distance of 1 nm between the particles.*

The DLVO calculations clearly elucidate that vdW attraction forces exceed the electrostatic repulsion for metallic Ni particles and that the presence of a surface oxide or a biocorona must be sufficiently thick (>9 nm) for the electrostatic forces to play a role.

REFERENCES

1. Benetti F, Bregoli L, Olivato I, Sabbioni E. Effects of metal(loid)-based nanomaterials on essential element homeostasis: The central role of nanometallomics for nanotoxicology. Metallomics. 2014;6(4):729-47. doi: 10.1039/C3MT00167A.
2. Auffan M, Rose J, Wiesner MR, Bottero J-Y. Chemical stability of metallic nanoparticles: A parameter controlling their potential cellular toxicity in vitro. Environmental Pollution. 2009;157(4):1127-33. doi: 10.1016/j.envpol.2008.10.002.
3. Casals E, Gonzalez E, Puntes VF. Reactivity of inorganic nanoparticles in biological environments: insights into nanotoxicity mechanisms. Journal of Physics D: Applied Physics. 2012;45(44):443001. doi: 10.1088/0022-3727/45/44/443001.
4. Janusz W. Electrical Double-Layer at Oxide-Solution Interfaces. In: Ponisseril S, editor. Encyclopedia of Surface and Colloid Science. 2. Boca Raton, FL, USA: CRC Press; 2006.
5. Lyklema J, de Keizer A. 3 - Electric Double Layers. In: Lyklema J, editor. Fundamentals of Interface and Colloid Science. 2: Academic Press; 1995. p. 3-1-3-232.
6. Israelachvili JN. Chapter 14 - Electrostatic Forces between Surfaces in Liquids. In: Israelachvili JN, editor. Intermolecular and Surface Forces (Third Edition). San Diego: Academic Press; 2011. p. 291-340.
7. Ohshima H. Electrokinetic Behaviour of Particles: Theory. In: Ponisseril S, editor. Encyclopedia of Surface and Colloid Science. 2. Boca Raton, FL, USA: CRC Press; 2006.
8. Russel WB, Saville DA, Schowalter WR. Colloidal Dispersions. Cambridge: Cambridge University Press; 1989.
9. Evans DF, Wennerström H. The Colloidal Domain: Where Physics, Chemistry, Biology, and Technology Meet. Second Edition ed: VCH Publishers; 1994.
10. Mei N, Hedberg J, Odnevall Wallinder I, Blomberg E. Influence of Biocorona Formation on the Transformation and Dissolution of Cobalt Nanoparticles under Physiological Conditions. ACS Omega. 2019;4(26):21778-91. doi: 10.1021/acsomega.9b02641.
11. Lin S, Mortimer M, Chen R, Kakinen A, Riviere JE, Davis TP, et al. NanoEHS beyond toxicity – focusing on biocorona. Environmental Science: Nano. 2017;4(7):1433-54. doi: 10.1039/C6EN00579A.
12. Zhang P, Cao M, Chetwynd AJ, Faserl K, Abdolahpur Monikh F, Zhang W, et al. Analysis of nanomaterial biocoronas in biological and environmental surroundings. Nature Protocols. 2024;19(10):3000-47. doi: 10.1038/s41596-024-01009-8.
13. Skoglund S, Hedberg J, Yunda E, Godymchuk A, Blomberg E, Odnevall Wallinder I. Difficulties and flaws in performing accurate determinations of zeta potentials of metal nanoparticles in complex solutions—Four case studies. PLOS ONE. 2017;12(7):e0181735. doi: 10.1371/journal.pone.0181735.
14. Ostolska I, Wiśniewska M. Application of the zeta potential measurements to explanation of colloidal Cr2O3 stability mechanism in the presence of the ionic polyamino acids. Colloid and Polymer Science. 2014;292(10):2453-64. doi: 10.1007/s00396-014-3276-y.
15. Hunter RJ. Introduction to modern colloid science. Oxford: Oxford Univ. Press; 1993.
16. Winzor DJ. Determination of the net charge (valence) of a protein: a fundamental but elusive parameter. Analytical Biochemistry. 2004;325(1):1-20. doi: 10.1016/j.ab.2003.09.035.
17. Dukhin AS, Parlia S. Measuring zeta potential of protein nano-particles using electroacoustics. Colloids and Surfaces B: Biointerfaces. 2014;121:257-63. doi: 10.1016/j.colsurfb.2014.02.048.
18. Delgado AV, González-Caballero F, Hunter RJ, Koopal LK, Lyklema J. Measurement and interpretation of electrokinetic phenomena. Journal of Colloid and Interface Science. 2007;309(2):194-224. doi: 10.1016/j.jcis.2006.12.075.
19. Doane TL, Chuang C-H, Hill RJ, Burda C. Nanoparticle ζ -Potentials. Accounts of Chemical Research. 2012;45(3):317-26. doi: 10.1021/ar200113c.
20. Hunter RJ. Chapter 2 - Charge and Potential Distribution at Interfaces. In: Hunter RJ, editor. Zeta Potential in Colloid Science: Academic Press; 1981. p. 11-58.

21. Lyons-Darden T, Heim KE, Han L, Haines L, Sayes CM, Oller AR. Bioaccessibility of Metallic Nickel and Nickel Oxide Nanoparticles in Four Simulated Biological Fluids. Nanomaterials. 2024;14(10):877. doi: 10.3390/nano14100877.
22. Israelachvili JN. Chapter 13 - Van der Waals Forces between Particles and Surfaces. In: Israelachvili JN, editor. Intermolecular and Surface Forces (Third Edition). San Diego: Academic Press; 2011. p. 253-89.
23. Min Y, Akbulut M, Kristiansen K, Golan Y, Israelachvili J. The role of interparticle and external forces in nanoparticle assembly. Nature Materials. 2008;7(7):527-38. doi: 10.1038/nmat2206.
24. Tokunaga TK. DLVO-Based Estimates of Adsorbed Water Film Thicknesses in Geologic CO2 Reservoirs. Langmuir. 2012;28(21):8001-9. doi: 10.1021/la2044587.
25. Chan DYC, Pashley RM, White LR. A simple algorithm for the calculation of the electrostatic repulsion between identical charged surfaces in electrolyte. Journal of Colloid and Interface Science. 1980;77(1):283-5. doi: 10.1016/0021-9797(80)90445-2.
26. Tolias P. Retarded room temperature Hamaker coefficients between bulk elemental metals. Surface Science. 2022;723:122123. doi: 10.1016/j.susc.2022.122123.
27. Pradhan S, Hedberg J, Blomberg E, Wold S, Odnevall Wallinder I. Effect of sonication on particle dispersion, administered dose and metal release of non-functionalized, non-inert metal nanoparticles. Journal of Nanoparticle Research. 2016;18(9):285. doi: 10.1007/s11051-016-3597-5.
28. Ninham BW, Parsegian VA. van der Waals Forces across Triple-Layer Films. The Journal of Chemical Physics. 1970;52(9):4578-87. doi: 10.1063/1.1673689.
